# E. H. Warner

**Published:** 1905-06

**Authors:** 


### E. H. WARNER.

Born at Winchester in 1856, educated at Sherborne and Switzerland, he received his medical training at Edinburgh, where he graduated in 1883. Shortly after qualifying he was appointed Resident Medical Assistant to the Lancaster County Asylum, and subsequently, in 1884, he was elected to the post of Assistant at the Bristol Asylum at Fishponds. After two years he relinquished this to undertake private practice at Barton Hill, Bristol, where he worked continuously until his health broke down. He first complained in August, 1904, and it was then found that he was suffering from pleurisy with effusion. He was advised to place himself under sanatorium treatment at Ringwood, as a precautionary measure, and there he improved so wonderfully in health as to justify a hope that he might recover, but in the early part of this year he broke down again and developed further effusion, thickened pleura, and pericarditis. He gradually sank and died of heart failure on April 17th, 1905.

He will be very much missed in his own circle; and the great respect in which he was held was evidenced by the presence of so large a congregation of his old patients and co-parishioners in the church of Holy Trinity, who listened to a telling and sympathetic address from the lips of the Rev. A. Ford, who was vicar when Dr. Warner was churchwarden a few years ago.

Dr. Warner was a man of the sweetest disposition, genial and kindly. He was an excellent practitioner, and his patients could always look upon him as their friend. Many will grieve at his death at so early an age as forty-eight.

